# Developing an Indirect ELISA for the Detection of African Swine Fever Virus Antibodies Using a Tag-Free p15 Protein Antigen

**DOI:** 10.3390/v15091939

**Published:** 2023-09-16

**Authors:** Zhi Wu, Huipeng Lu, Dewei Zhu, Jun Xie, Fan Sun, Yan Xu, Hua Zhang, Zhijun Wu, Wenlong Xia, Shanyuan Zhu

**Affiliations:** 1 Jiangsu Key Laboratory for High-Tech Research and Development of Veterinary Biopharmaceuticals, Jiangsu Agri-Animal Husbandry Vocational College, Taizhou 225300, China; yzwuzhi@163.com (Z.W.); luhuipeng66@126.com (H.L.); xjjstz2018@163.com (J.X.); 18860891675@163.com (F.S.); xy18189517254@163.com (Y.X.); 2 Yancheng Engineering Research Center of Animal Biologics, School of Marine and Biological Engineering, Yancheng Teachers University, Yancheng 224007, China; zhudw@yctu.edu.cn; 3 School of Pharmacy, Yancheng Teachers University, Yancheng 224007, China; zhangh01@yctu.edu.cn (H.Z.); wuzj@yctu.edu.cn (Z.W.); 4 Jiangsu Province Engineering Research Center of Tumor Targeted Nano Diagnostic and Therapeutic Materials, Yancheng Teachers University, Yancheng 224007, China

**Keywords:** African swine fever virus, p15 protein, tag-free antigen, indirect ELISA

## Abstract

African swine fever (ASF) is one of the most severe diseases caused by the ASF virus (ASFV), causing massive economic losses to the global pig industry. Serological tests are important in ASF epidemiological surveillance, and more antigen targets are needed to meet market demand for ASFV antibody detection. In the present study, ASFV p15 protein was fusion-expressed in *Escherichia coli* (*E. coli*) with elastin-like polypeptide (ELP), and the ELP-p15 protein was purified using a simple inverse transition cycling (ITC) process. The ELP tag was cleaved off using tobacco etch virus protease (TEVp), resulting in a tag-free p15 protein. Western blot analysis demonstrated that the p15 protein reacted strongly with ASFV-positive serum. The p15 protein was used as a coating antigen in an indirect ELISA (iELISA) for detecting ASFV antibodies. The p15-iELISA method demonstrated high specificity to ASFV-positive sera, with a maximum detection dilution of 1:1600. Moreover, the method exhibited good reproducibility, with less intra-assay and inter-assay CV values than 10%. Therefore, p15-iELISA offers a novel approach for accurately detecting ASFV antibodies with significant clinical application potential.

## 1. Introduction

African swine fever (ASF) is a highly contagious infectious disease caused by the African swine fever virus (ASFV). This virus can infect pigs of all breeds and ages, causing symptoms such as high fever and systemic organ hemorrhage, with mortality rates up to 100% [1]. Since its discovery in Kenya in 1921, ASF has spread to more than 60 countries worldwide [2]. Although ASFV does not infect humans or pose a threat to public health, it has been a major threat to the global swine industry, with significant social and economic impact. ASF was first introduced in China in August 2018 and quickly spread throughout the country, causing massive economic losses to the swine industry [3]. The World Organization for Animal Health (WOAH) has classified ASF as a notifiable animal disease, and the Chinese government classified it as a first-class animal infectious disease [4].

ASFV is a large double-stranded DNA virus with a genome size of approximately 170–193 kb encoding about 150–200 proteins, with the functions of more than half of these proteins remaining unknown [5,6]. ASFV primarily targets monocytes and macrophage cells in vivo, resulting in a slow antiviral response and persistent infection [7]. In addition, the virus has evolved various evasion strategies against host immunity, including the inhibition of interferon and inflammatory responses, as well as the regulation of apoptosis and autophagy [8,9,10,11]. The development of vaccines faces a significant challenge due to the unknown function of numerous ASFV proteins and the complex biological properties of infection. At present, the strategy for ASF prevention and control encompasses three aspects: early diagnosis, the elimination of infectious sources, and cutting off transmission routes. Thus, accurate and timely laboratory tests are critical for the surveillance and control of ASF.

Because no effective commercial vaccines or medicines against ASFV are currently available, the presence of specific antibodies in serum can be used as an indicator of virus infection [12]. ELISA is the most commonly used serological method because of its sensitivity, accuracy, simplicity, and economics [13,14]. Moreover, molecular biology techniques (such as PCR and fluorescence quantitative PCR) for ASFV nucleic acid detection are also widely used in many laboratories due to their advantages of high sensitivity and specificity [15,16]. However, these methods usually require a tedious nucleic acid extraction process, expensive equipment, and professional technicians. Additionally, naturally occurring lower virulent ASFV strains have been identified in China since 2020 [17]. These strains can cause nonlethal or subacute disease with intermittent virus shedding, which brings greater difficulty to the molecular-based early diagnosis methods. Although ELISA is usually less sensitive than PCR, it holds significant practical value owing to its simplicity of operation and convenient application for large-scale surveillance [18].

The WOAH has deemed ELISA as the first serological technique for ASF diagnosis [19]. There are mainly three commercial ELISA kits based on ASFV p32, p62, and p72 proteins (ID. Vet, Montpellier, France), p72 protein (Ingenasa, Madrid, Spain), and p30 protein (Svanova, Uppsala, Sweden) [20]. However, these kits are expensive and may not guarantee stable supply in practical application. Recently, several proteins have been identified as potential antigens for ASFV antibody detection, including CD2v, pB602L, and pp62 [20,21,22]. Previous studies revealed that the ASFV pp62 protein encoded by the *CP530R* gene is highly immunogenic and has been successfully used as an antigen in indirect ELISA (iELISA) for ASFV antibody detection [22]. pp62 protein, as a polyprotein precursor, is cleaved by S273R proteinase during virus maturation to form two major products, p15 protein, and p35 protein, which eventually contribute to the composition of the virion core–shell [5,23]. Thus, further research is required into the antigenic epitope with the potential for serological diagnosis in p15 and p35 proteins.

Elastin-like polypeptide (ELP) is a class of artificially synthesized pentapeptide polymers based on mammalian elastin with temperature-sensitive reversible phase transition characteristics [24]. The ELP fusion proteins can be purified through a separation process called inverse transition cycling (ITC) using temperature-controlled centrifugation, providing a simple and efficient strategy for target protein purification [25]. Tobacco etch virus protease (TEVp) is a common protease that recognizes the amino acid sequence ENLYFQ/G and has been widely used to cleave the junction between the fusion tag and the target protein [26,27].

The present study aimed to investigate whether the p15 protein can be used for serological diagnosis to detect ASFV-specific antibodies. ELP-fused recombinant p15 protein was expressed in the *E. coli* expression system and purified via ITC. To avoid the potential false positive signal due to ELP tag cross-reactivity with antibody/serum, TEVp was used to cleavage the ELP tag, resulting in a tag-free p15 protein. Subsequently, an iELISA method for detecting ASFV antibodies was developed using the p15 protein as an antigen, laying a foundation for developing ASFV early diagnosis kits and further researching the p15 protein.

## 2. Materials and Methods

### 2.1. Sera and Plasmid

We collected and preserved swine-positive sera against porcine circovirus type 2 (PCV2), porcine reproductive and respiratory syndrome virus (PRRSV), porcine pseudorabies virus (PRV), classical swine fever virus (CSFV), and foot-and-mouth disease virus (FMDV) in our laboratory. ASFV-positive (*n* = 12) and ASFV-negative (*n* = 1) standard sera were purchased from the China National Center for Veterinary Culture Collection (CVCC, Beijing, China). All the ASFV-positive standard serum samples were collected from pigs 30 days after artificial challenge with the CD2v gene-deleted ASFV strain Hub/2019 and tested positive for ASFV using an indirect immunofluorescence assay (IFA) conducted by the CVCC. Clinical swine serum samples (*n* = 102) were collected from pig farms in Jiangsu Province of China in 2015–2017 (before the ASF outbreak in China). The ASFV-negative standard serum and all clinical samples were tested negative for ASFV using commercial fluorescence quantitative PCR (FQ-PCR) (Lijian Bio-Tech Co., Ltd., Qingdao, China) and ELISA (Ingenasa, Madrid, Spain) kits.

The recombinant expression plasmid pET-ELP was developed in our previous studies [28]. The ASFV *CP530R* gene sequence (GenBank accession No. QBH90582.1) was adapted to *E. coli* codon usage using the Java Codon Adaptation Tool (https://www.prodoric.de/JCat, accessed on 6 January 2023), and then synthesized and inserted into pUC57 vector by Sangon Biotech Co., Ltd. (Shanghai, China), resulting in plasmid pUC57-CP530R.

### 2.2. Preparation of Tag-Free p15 Protein Antigen

#### 2.2.1. Construction of Recombinant Expression Vector pELP-p15

The antigenic index of the p15 protein was analyzed using the Protean program of DNASTAR software (Lasergene v7.0, Inc., Madison, WI, USA). The full-length p15 coding sequence was amplified with PCR using pUC57-CP530R plasmid as a template with primers F (5′-GCCGGGCGGGCTGGTGAGCTCCGAAAACCTGTACTTCCAGGGTCCCAGCAACATGAAGCAG-3′) and R (5′-GTGGTGGTGGTGGTGCTCGAGTTAGCCCCCCCCCTCCTTCTT-3′). The coding sequence for the TEVp recognition site was introduced to the forward primer (underlined sequence). The PCR product was cloned into the pET-ELP vector using a ClonExpress II One-Step Cloning Kit (Vazyme Biotech Co., Ltd., Nanjing, China), yielding the recombinant expression vector pELP-p15.

#### 2.2.2. Protein Expression and Purification

The recombinant plasmid pELP-p15 was transformed into *E. coli* BL21 (DE3), and protein expression was induced with 0.2 mM IPTG at 20 °C for 16 h. The cells were harvested and disrupted via sonication for 10 min. Both supernatant and precipitation were collected and analyzed using 12% SDS-PAGE after centrifugation at 8000× *g* for 10 min at 4 °C. The ELP-p15 fusion protein was then purified using ITC, as described in our previous study [28]. First, 2 mL of bacterial lysate supernatant was incubated with 3 M (final concentration) of NaCl for 10 min at 22, 24, 26, 28, and 30 °C, respectively. The supernatant was then incubated with an equal volume of 3, 4, 5, or 6 M NaCl at the optimized temperature. The precipitated protein was analyzed using 12% SDS-PAGE after each mixture solution was centrifuged at 12,000× *g* for 5 min at room temperature.

#### 2.2.3. p15 Protein Recovery via Protease Cleavage

**Figure 1:**
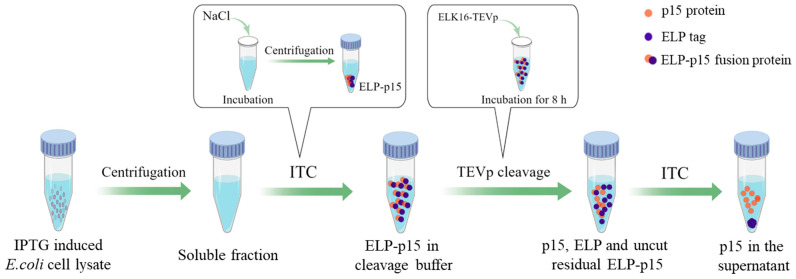
The process of tag-free p15 protein preparation. ITC, inverse transition cycling; ELP, Elastin-like polypeptide; TEVp, tobacco etch virus protease. The figure was drawn using the online software Figdraw (https://www.figdraw.com/, accessed on 5 June 2023).

According to our previous study [29], the ELP tag was removed from the ELP-p15 fusion protein using the self-assembling peptide ELK16-tagged TEVp (ELK16-TEVp). Briefly, the precipitated ELP-p15 fusion protein was suspended in TEVp cleavage buffer (50 mM Tris-HCl, 0.5 mM EDTA, and 1 mM DTT, pH 8.0), and ELK16-TEVp was added at a final concentration of 100 μg/mL. The cleavage reaction was carried out at 30 °C for 8 h, followed by a 10 min centrifugation at 16,000× *g* at 4 °C to remove ELK16-TEVp. ITC removed the cut ELP tag and uncut residual ELP-p15 fusion protein with the above-optimized conditions, and the supernatant was collected (Figure 1) and dialyzed with PBS (pH 7.2) at 4 °C for 2 h. Therefore, tag-free p15 protein was obtained and identified using 12% SDS-PAGE and Western blot. One of the ASFV-positive standard sera with a dilution of 1:2000 was used as the primary antibody for Western blot, and goat anti-swine IgG (Solarbio Life Sciences Co., Ltd., Beijing, China) with a dilution of 1:5000 was used as the secondary antibody.

### 2.3. Establishment of Indirect ELISA (iELISA) Method

#### 2.3.1. Checkerboard Titration

The tag-free p15 protein antigen concentration and serum dilution were optimized using checkerboard titration. Briefly, the p15 antigen was coated on 96-well plates at 16.0, 8.0, 4.0, 2.0, 1.0, and 0.5 μg/mL. ASFV-positive and ASFV-negative standard sera were diluted at 1:100, 1:200, and 1:400. The ASFV-positive serum had been determined to show a titer of 1:4000 using an indirect immunofluorescence assay (IFA) conducted by the CVCC. Each test was repeated three times, and the average results of OD_450_ were calculated. The best antigen coating concentration and sera dilution were determined by having the highest P/N value (OD_450_ ratio between positive and negative sera).

#### 2.3.2. Optimization of Reaction Conditions

Other reaction conditions were optimized based on the best antigen coating concentration and sera dilution. Different coating buffers (0.05 M carbonate at pH 9.6, PBS at pH 7.2, and 0.05 M Tris-HCl at pH 8.5) and blocking buffers (PBS with 5% albumin, 5% skim milk, 5% BSA, 1% gelatin, or 1% alginate) were optimized. The optimal secondary antibody (Abcam China Co. Ltd., Shanghai, China) dilution (1:5000, 1:10,000, 1:15,000, and 1:20,000) was then selected.

#### 2.3.3. Determination of Cut-Off Value

To determine the cut-off value, 64 ASFV-negative sera were randomly selected from the validated negative clinical swine serum samples and detected under optimal working conditions. The mean average ($\bar{x}$) and standard deviation (SD) of the OD_450_ values of the 64 samples were calculated. The cut-off value of the iELISA method was determined to be $\bar{x}$ + 3SD.

#### 2.3.4. Specificity and Sensitivity Tests

The ASFV-negative standard serum and swine serum samples positive for PCV2, PRRSV, PRV, CSFV, and FMDV were used to evaluate the specificity of p15-iELISA. The ASFV-positive standard serum sample was used as a positive control. ASFV-positive standard serum (the same as that used for checkerboard titration) was diluted 1:100, 1:200, 1:400, 1:800, 1:1600, 1:3200, 1:6400, and 1:12,800. The serial dilutions were used to determine the sensitivity of p15-iELISA.

#### 2.3.5. Reproducibility Test

Six serum samples (five were ASFV-positive, and one was ASFV-negative) were selected randomly. Each sample was detected in triplicate using p15-iELISA in one batch to test the intra-assay variation. In addition, each sample was assayed in three batches separately to determine the inter-assay variation. The intra-assay and inter-assay coefficients of variation (CVs) were calculated.

#### 2.3.6. Comparison with the Commercial Kit

A total of 114 swine serum samples (12 ASFV-positive standard sera + 102 ASFV-negative clinical sera) were detected using the p15-iELISA developed in the present study. They were also detected using a commercial ASFV antibody detection kit (Ingenasa, Madrid, Spain) for comparison.

## 3. Results

### 3.1. p15 Protein Antigen Preparation

#### 3.1.1. Expression of ELP-p15 Fusion Protein

**Figure 2:**
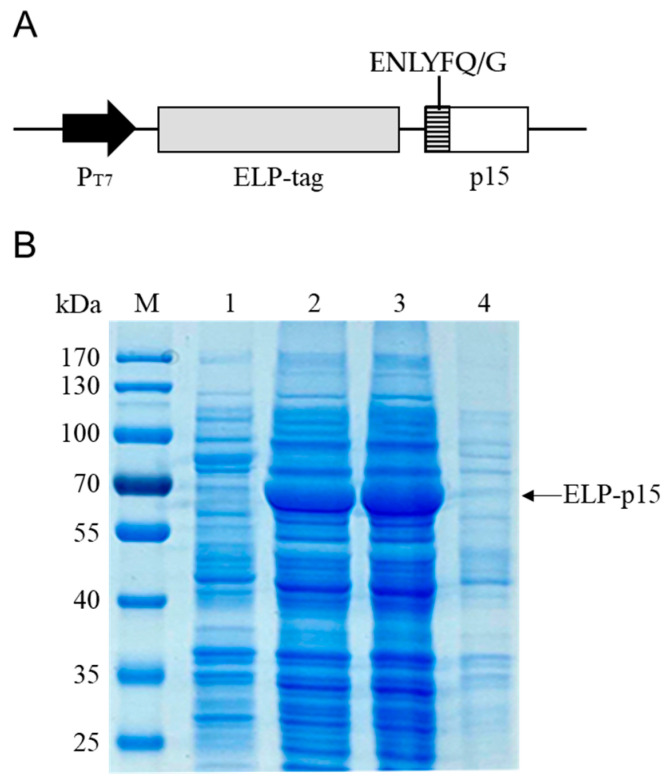
(**A**) The schematic structure of pET-ELP-p15 expression vector. ENLYFQ/G indicates the TEVp recognition site; (**B**) SDS-PAGE analysis of the expression of ELP-p15 fusion protein. Lane 1: uninduced pET-ELP-p15 recombinant *E. coli* cell lysate; Lane 2: IPTG-induced *E. coli* cell lysate; Lane 3: soluble fraction of IPTG-induced *E. coli* cell lysate; Lane 4: insoluble fraction of IPTG-induced *E. coli* cell lysate.

The ASFV p15 protein coding sequence was amplified and cloned into the pET-ELP vector (Figure 2A). The ELP-p15 fusion protein expression was induced in *E. coli* BL21 (DE3) with IPTG. SDS-PAGE analysis revealed that an expected 62.1 kDa extra band (44.9 kDa ELP + 17.2 kDa p15) was present after IPTG induction compared with uninduced recombinant bacteria lysate. Moreover, the expressed ELP-p15 protein was soluble, as evidenced by its presence in the cell lysate supernatant (Figure 2B).

#### 3.1.2. Purification of ELP-p15 Fusion Protein

**Figure 3:**
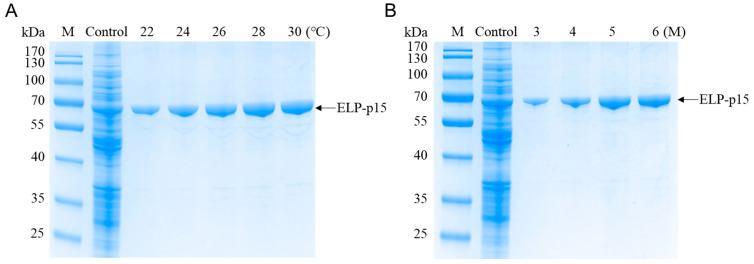
(**A**) Purification of ELP-p15 fusion protein under different ITC temperatures; (**B**) purification of ELP-p15 fusion protein using ITC with different NaCl concentrations. ITC, inverse transition cycling.

First, ELP-p15 protein was purified using ITC under different transition temperatures with 3.0 M NaCl. SDS-PAGE analysis revealed that the recovery of ELP-p15 protein increased gradually with increasing temperature, with the maximum yield achieved at 28–30 °C (Figure 3A). Then, ITC was performed with different concentrations of NaCl at 28 °C, and the result demonstrated a successful recovery of ELP-p15 proteins with 5–6 M NaCl with a similar yield (Figure 3B). Therefore, the optimal ITC incubation temperature and salt concentration for ELP-p15 protein purification were 28 °C and 5 M NaCl.

#### 3.1.3. Recovery and Identification of Tag-Free p15 Protein

**Figure 4:**
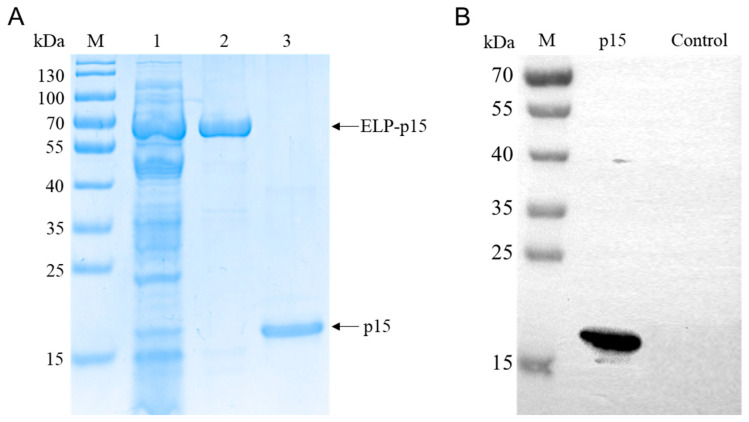
(**A**) SDS-PAGE analysis for the cleavage of ELP-p15 fusion protein. Lane 1: clarified *E. coli* cell lysate before ITC purification; Lane 2: ELP-p15 purified using ITC; Lane 3: p15 cleaved from ELP-p15 using ELK16-TEVp; (**B**) Western blot analysis of p15 protein using the ASFV-positive serum. ITC, inverse transition cycling.

The ELP tag was removed from the ELP-p15 fusion protein via ELK16-TEVp cleavage. After cleavage, SDS-PAGE analysis revealed a single p15 protein band with the expected size of 17.2 kDa (Figure 4A). Furthermore, the Western blot analysis revealed that ASFV-positive standard serum enabled the detection of the tag-free p15 protein (Figure 4B), indicating that this protein could be used as an iELISA antigen.

### 3.2. p15-iELISA Method Establishment and Evaluation

#### 3.2.1. Working Conditions for p15-iELISA

**Figure 5:**
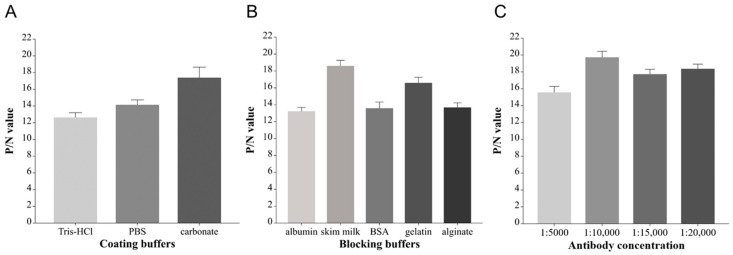
(**A**) Optimization of coating buffer. The 0.05 M carbonate (pH 9.6) was selected as the best coating buffer; (**B**) optimization of blocking buffer. The 5% skim milk in PBS was selected as the optimal blocking buffer; (**C**) determination of secondary antibody concentration. A dilution of 1:10,000 was selected as the best secondary antibody concentration.

The optimal concentration of p15 protein antigen and serum dilution was selected using checkerboard titration. The highest P/N value was achieved when the antigen coating concentration was 2 µg/mL, and the serum dilution was 1:200, as shown in Table 1. Other reaction conditions were then optimized. The best coating buffer was 0.05 M carbonate (pH 9.6), and 5% skim milk in PBS was the optimal blocking buffer. The best secondary antibody dilution was 1:10,000 (Figure 5).

#### 3.2.2. Cut-Off Value Determination

**Figure 6:**
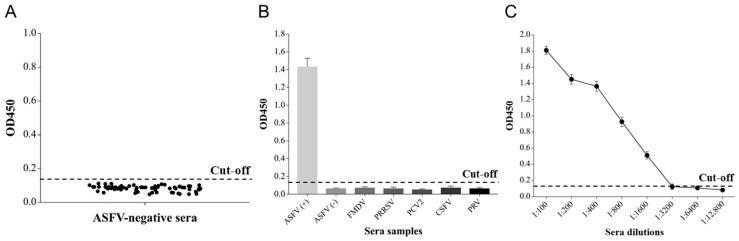
(**A**) Determination of the cut-off value using ASFV-negative serum samples; (**B**) specificity of p15-iELISA for ASFV antibody detection; (**C**) sensitivity test of p15-iELISA.

The cut-off value was determined by detecting 64 ASFV-negative sera with the p15-iELISA. The average OD_450_ value ($\bar{x}$) of these negative samples was 0.083, with an SD of 0.017. Therefore, the cut-off value was calculated to be 0.133 ($\bar{x}$ + 3SD) (Figure 6A). When the OD_450_ value of the tested serum sample was ≥0.133, it was positive; when it was <0.133, it was negative.

#### 3.2.3. Specificity Test

The specificity of the p15-iELISA was tested using the ASFV-negative serum and positive sera against other porcine viruses. Only ASFV-positive control serum was detected positive, while all other sera were negative (Figure 6B), indicating that p15-iELISA had no cross-reaction with other anti-virus swine sera.

#### 3.2.4. Sensitivity Test

The sensitivity of the p15-iELISA was tested using serial dilutions (1:100 to 1:12,800) of the ASFV-positive standard serum sample. The highest positive dilution detected with the assay was 1:1600, as presented in Figure 6C.

#### 3.2.5. Reproducibility Test

Table 2 indicates that the intra-assay CV was 1.68–2.88%, and the inter-assay CV was 1.01–4.04%. The CV values were <10%, indicating a good reproducibility of p15-iELISA.

#### 3.2.6. Comparison with the Commercial Kit

The established p15-iELISA was used to detect 114 swine serum samples, and a commercial ASFV antibody detection kit was used as a standard for comparison. The positive/negative samples of p15-iELISA and the commercial kit were both 12/102, and the coincidence rate between the two methods was 100% (Table 3).

## 4. Discussion

Despite numerous efforts to combat ASF, it remains one of the most serious diseases affecting the global swine industry. ASFV was first introduced to China in 2018 and was identified as a highly virulent genotype II strain [3]. Since 2020, less virulent genotype II strains with mutations, deletions, or insertions have been isolated in China compared with the original strain, resulting in a complex epidemic [17]. Early detection is critical to its control and prevents the spread of ASFV. Several techniques have been used for ASFV detection in early diagnosis, including molecular approaches such as PCR and fluorescence quantitative PCR (FQ-PCR), as well as immunological methods such as colloidal gold rapid strip and ELISA [15,16,22,30]. ELISA is one of these methods used in continuous epidemiological surveillance of ASF.

Commercial ELISA kits for ASFV antibody detection primarily target the p30, p54, and p72 proteins [20]. Considering the importance of serological tests in ASF epidemiological investigation and surveillance, additional antigen targets are urgently needed to meet market demand. As a structural protein, the pp62 protein has high antigenicity, and ASFV-infected pigs can exhibit high antibody titers [5,31]. In a previous study, pp62 protein expressed with the baculovirus expression system was used as an antigen to develop an iELISA for ASFV antibody detection with better sensitivity and specificity than p32- and p54-based iELISA [32]. Further study revealed that the pp62 protein expressed using the *E. coli* prokaryotic expression system exhibited strong antigenicity, and an iELISA based on this antigen demonstrated excellent performance [22]. However, pp62 is a polyprotein precursor cleaved into two major proteins (p35 and p15) during viral capsid assembly [5]. We identified that the p15 protein had a high antigenic index predicted using the Protean program of DNASTAR software (Figure S1), and its amino acid sequence was highly conserved among the different ASFV strains (Figure S2). The present study investigated its potential use as an antigen for ASFV serological detection.

As the most common recombinant protein expression system, the *E. coli* prokaryotic expression system has the advantages of being simple, rapid, economical, and high-level expression. Recombinant proteins expressed with the prokaryotic expression system are always fused with a His or GST tag to facilitate subsequent affinity purification. Notably, some subunit protein vaccines for porcine infectious disease used His- or GST-tagged fusion protein as antigens [33,34], which may induce anti-tagged antibodies in vaccinated porcine sera. Moreover, most antigen proteins in ELISA methods for ASFV antibody detection contain tag proteins [20,21,22,35], which may cause false-positive results. In order to eliminate the potential false positives, the present study prepared a tag-free p15 protein antigen. Previous studies demonstrated ELP as an efficient tag for target protein purification [28]. The present study used ELP as a fusion tag for p15 protein expression and purification. The recombinant ELP-p15 protein was purified using a simple temperature-controlled centrifugation process without using expensive affinity columns. To cut off the ELP tag, a TEVp recognition site was first introduced between ELP and p15 coding sequences. Therefore, the purified ELP-p15 fusion protein could be cleaved by TEVp, resulting in a tag-free p15 protein. Furthermore, the p15 protein reacted significantly with ASFV-positive swine serum, indicating good antigenicity. This purification and cleavage strategy provides a novel idea for producing other tag-free proteins.

An iELISA method for ASFV antibody detection method was subsequently established and optimized using the p15 protein coating antigen. The developed p15-iELISA was so specific that no cross-reaction with anti-sera from common porcine viruses such as CSFV, PRRSV, FMDV, PCV2, and PRV was observed. Moreover, the iELISA enabled the detection of the ASFV-positive standard serum at the highest dilution of 1:1600, comparable to the pp62 antigen-based iELISA previously reported in the literature [22]. In addition, the iELISA demonstrated good reproducibility, with both intra-assay and inter-assay CVs of less than 10%. Furthermore, the coincidence rate between p15-iELISA and the commercial ASFV antibody detection kit was 100%, indicating a significant clinical potential.

In China, live ASFV manipulations and animal infection experiments must be conducted in animal biosafety level 3 (ABSL-3) laboratories. Considering this situation, there are some aspects of p15-iELISA that need to be improved. Firstly, the 12 ASFV-positive serum samples used in our study were all collected from pigs injected with the same ASFV strain Hub/2019, and we just used one of the sera to test the sensitivity of the p15-iELISA. In future work, we intend to utilize more sera against other ASFV strains to evaluate the sensitivity and clinical performance of the p15-iELISA. Moreover, the kinetic of the antibody response in serum to ASFV p15 is unclear, which may impede the understanding of the role of p15 as a marker for ASFV serological studies. This point will be the focus of our further research.

## 5. Conclusions

In the present study, we obtained a tag-free p15 protein of ASFV using ELP tag purification and TEVp cleavage. An iELISA method for ASFV antibody detection based on the p15 protein coating antigen was developed with good specificity and reproducibility. The p15-iELISA method appears to be a reliable tool for ASFV antibody detection, laying a foundation for the epidemiological investigation and prevention of ASF in the future.

## Figures and Tables

**Table 1:** Determination of optimal antigen concentration and serum dilution for p15-iELISA.

Serum Dilution		Concentrations of p15 Protein (μg/mL)					
		16	8	4	2	1	0.5
1:100	P	1.716 ± 0.017	1.578 ± 0.016	1.531 ± 0.026	1.517 ± 0.019	1.519 ± 0.020	1.426 ± 0.034
	N	0.116 ± 0.005	0.098 ± 0.002	0.102 ± 0.003	0.098 ± 0.003	0.093 ± 0.004	0.115 ± 0.002
	P/N	14.8	16.1	15.0	15.5	16.3	12.4
1:200	P	1.587 ± 0.022	1.524 ± 0.016	1.488 ± 0.019	1.41 ± 0.017	1.316 ± 0.016	1.227 ± 0.020
	N	0.097 ± 0.003	0.098 ± 0.002	0.102 ± 0.005	0.071 ± 0.001	0.07 ± 0.002	0.07 ± 0.002
	P/N	16.4	15.6	14.6	19.9	18.8	17.5
1:400	P	1.458 ± 0.029	1.407 ± 0.032	1.346 ± 0.031	1.256 ± 0.013	1.175 ± 0.024	0.851 ± 0.014
	N	0.088 ± 0.004	0.083 ± 0.003	0.081 ± 0.002	0.078 ± 0.002	0.077 ± 0.003	0.079 ± 0.001
	P/N	16.6	17.0	16.6	16.1	15.3	10.8

P, positive; N, negative. Results are displayed as mean ± SD.

**Table 2:** Reproducibility test of the p15-iELISA.

Samples	Intra-Assay		Inter-Assay	
	OD_450_ (Mean ± SD)	CV	OD_450_ (Mean ± SD)	CV
1	0.835 ± 0.018	2.16%	0.852 ± 0.023	2.70%
2	1.215 ± 0.035	2.88%	1.261 ± 0.051	4.04%
3	0.654 ± 0.011	1.68%	0.679 ± 0.020	2.95%
4	0.111 ± 0.002	1.80%	0.099 ± 0.001	1.01%
5	0.689 ± 0.013	1.89%	0.658 ± 0.021	3.19%
6	0.945 ± 0.022	2.33%	1.017 ± 0.036	3.54%

**Table 3:** The reproducibility test of p15-iELISA.

Methods		Commercial Kit			Coincidence Rate
		Positive	Negative	Total	
p15-iELISA	Positive	12	0	12	100%
	Negative	0	102	102	
	Total	12	102	114	

## Data Availability

The data presented in this study are available on request from the corresponding author.

## Supplementary Materials

The following supporting information can be downloaded at: https://www.mdpi.com/article/10.3390/v15091939/s1, Figure S1: Antigenic index of p15 protein predicted using the Protean program of DNASTAR software; Figure S2: Amino acid sequences analysis of p15 protein among different ASFV strains.
