# Supplementary material for: Developing an Indirect ELISA for the Detection of African Swine Fever Virus Antibodies Using a Tag-Free p15 Protein Antigen
Source: Viruses. 2023 Sep 16;15(9):1939. doi: 10.3390/v15091939 (PMC10534517; doi:10.3390/v15091939)
Supplement: Supplementary file 1 [file viruses-2524635-supplementary.pdf › viruses-2524635-supplementary.pdf]

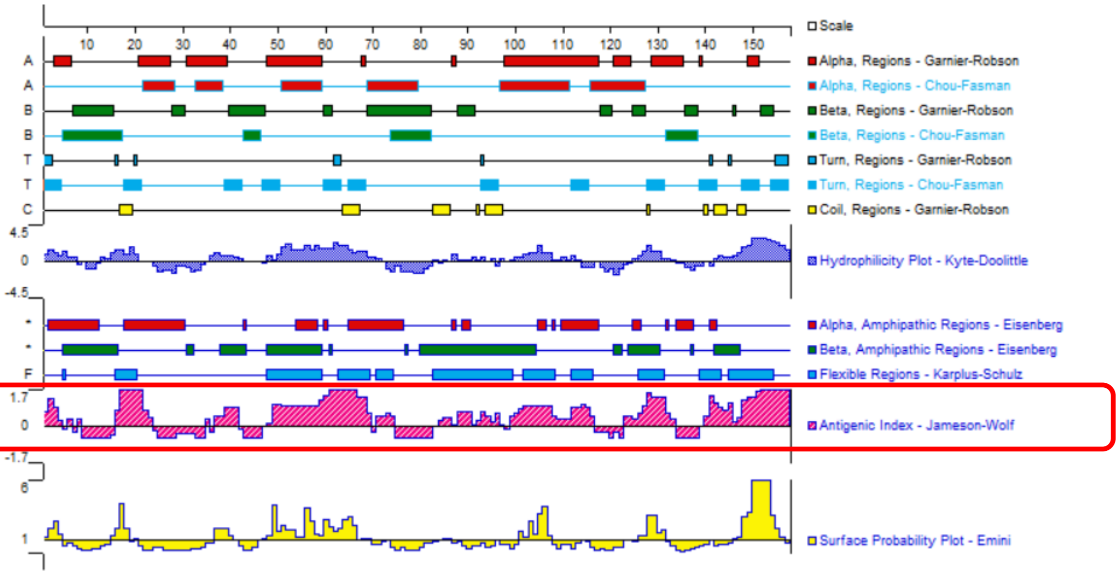

Figure S1. Antigenic index of p15 protein predicted by the Protean program of DNASTAR software.

Percent Identity

Divergence

	1	2	3	4	5	6	7	8	9	10	11	12	13	14	15	16	17	18	19	20	21	22	23	24	25	26	27	28	29	30	31	32	33	34	35	36	37		
1		100.0	100.0	100.0	100.0	100.0	100.0	100.0	100.0	100.0	100.0	100.0	100.0	100.0	100.0	100.0	100.0	100.0	100.0	99.8	100.0	100.0	100.0	100.0	100.0	100.0	100.0	100.0	100.0	99.8	100.0	100.0	100.0	100.0	100.0	99.8	100.0	1	QOY24199.1
2	0.0		100.0	100.0	100.0	100.0	100.0	100.0	100.0	100.0	100.0	100.0	100.0	100.0	100.0	100.0	100.0	100.0	100.0	99.8	100.0	100.0	100.0	100.0	100.0	100.0	100.0	100.0	100.0	99.8	100.0	100.0	100.0	100.0	100.0	99.8	100.0	2	QDL88125.1
3	0.0	0.0		100.0	100.0	100.0	100.0	100.0	100.0	100.0	100.0	100.0	100.0	100.0	100.0	100.0	100.0	100.0	100.0	99.8	100.0	100.0	100.0	100.0	100.0	100.0	100.0	100.0	100.0	99.8	100.0	100.0	100.0	100.0	100.0	99.8	100.0	3	UVI02157.1
4	0.0	0.0	0.0		100.0	100.0	100.0	100.0	100.0	100.0	100.0	100.0	100.0	100.0	100.0	100.0	100.0	100.0	100.0	99.8	100.0	100.0	100.0	100.0	100.0	100.0	100.0	100.0	100.0	99.8	100.0	100.0	100.0	100.0	100.0	99.8	100.0	4	QTE18772.1
5	0.0	0.0	0.0	0.0		100.0	100.0	100.0	100.0	100.0	100.0	100.0	100.0	100.0	100.0	100.0	100.0	100.0	100.0	99.8	100.0	100.0	100.0	100.0	100.0	100.0	100.0	100.0	100.0	99.8	100.0	100.0	100.0	100.0	100.0	99.8	100.0	5	UNZ12441.1
6	0.0	0.0	0.0	0.0	0.0		100.0	100.0	100.0	100.0	100.0	100.0	100.0	100.0	100.0	100.0	100.0	100.0	100.0	99.8	100.0	100.0	100.0	100.0	100.0	100.0	100.0	100.0	100.0	99.8	100.0	100.0	100.0	100.0	100.0	99.8	100.0	6	UMA73198.1
7	0.0	0.0	0.0	0.0	0.0	0.0		100.0	100.0	100.0	100.0	100.0	100.0	100.0	100.0	100.0	100.0	100.0	100.0	99.8	100.0	100.0	100.0	100.0	100.0	100.0	100.0	100.0	100.0	99.8	100.0	100.0	100.0	100.0	100.0	99.8	100.0	7	QOY24400.1
8	0.0	0.0	0.0	0.0	0.0	0.0	0.0		100.0	100.0	100.0	100.0	100.0	100.0	100.0	100.0	100.0	100.0	100.0	99.8	100.0	100.0	100.0	100.0	100.0	100.0	100.0	100.0	100.0	99.8	100.0	100.0	100.0	100.0	100.0	99.8	100.0	8	QUQ60559.1
9	0.0	0.0	0.0	0.0	0.0	0.0	0.0	0.0		100.0	100.0	100.0	100.0	100.0	100.0	100.0	100.0	100.0	100.0	99.8	100.0	100.0	100.0	100.0	100.0	100.0	100.0	100.0	100.0	99.8	100.0	100.0	100.0	100.0	100.0	99.8	100.0	9	UYE97669.1
10	0.0	0.0	0.0	0.0	0.0	0.0	0.0	0.0	0.0		100.0	100.0	100.0	100.0	100.0	100.0	100.0	100.0	100.0	99.8	100.0	100.0	100.0	100.0	100.0	100.0	100.0	100.0	100.0	99.8	100.0	100.0	100.0	100.0	100.0	99.8	100.0	10	AZP54160.1
11	0.0	0.0	0.0	0.0	0.0	0.0	0.0	0.0	0.0	0.0		100.0	100.0	100.0	100.0	100.0	100.0	100.0	100.0	99.8	100.0	100.0	100.0	100.0	100.0	100.0	100.0	100.0	100.0	99.8	100.0	100.0	100.0	100.0	100.0	99.8	100.0	11	WGM83282.1
12	0.0	0.0	0.0	0.0	0.0	0.0	0.0	0.0	0.0	0.0	0.0		100.0	100.0	100.0	100.0	100.0	100.0	100.0	99.8	100.0	100.0	100.0	100.0	100.0	100.0	100.0	100.0	100.0	99.8	100.0	100.0	100.0	100.0	100.0	99.8	100.0	12	WFS78378.1
13	0.0	0.0	0.0	0.0	0.0	0.0	0.0	0.0	0.0	0.0	0.0	0.0		100.0	100.0	100.0	100.0	100.0	100.0	99.8	100.0	100.0	100.0	100.0	100.0	100.0	100.0	100.0	100.0	99.8	100.0	100.0	100.0	100.0	100.0	99.8	100.0	13	WEG42617.1
14	0.0	0.0	0.0	0.0	0.0	0.0	0.0	0.0	0.0	0.0	0.0	0.0	0.0		100.0	100.0	100.0	100.0	100.0	99.8	100.0	100.0	100.0	100.0	100.0	100.0	100.0	100.0	100.0	99.8	100.0	100.0	100.0	100.0	100.0	99.8	100.0	14	WBR50386.1
15	0.0	0.0	0.0	0.0	0.0	0.0	0.0	0.0	0.0	0.0	0.0	0.0	0.0	0.0		100.0	100.0	100.0	100.0	99.8	100.0	100.0	100.0	100.0	100.0	100.0	100.0	100.0	100.0	99.8	100.0	100.0	100.0	100.0	100.0	99.8	100.0	15	CAI4209689.1
16	0.0	0.0	0.0	0.0	0.0	0.0	0.0	0.0	0.0	0.0	0.0	0.0	0.0	0.0	0.0		100.0	100.0	100.0	99.8	100.0	100.0	100.0	100.0	100.0	100.0	100.0	100.0	100.0	99.8	100.0	100.0	100.0	100.0	100.0	99.8	100.0	16	UZT04484.1
17	0.0	0.0	0.0	0.0	0.0	0.0	0.0	0.0	0.0	0.0	0.0	0.0	0.0	0.0	0.0	0.0		100.0	100.0	99.8	100.0	100.0	100.0	100.0	100.0	100.0	100.0	100.0	100.0	99.8	100.0	100.0	100.0	100.0	100.0	99.8	100.0	17	BDC47329.1
18	0.0	0.0	0.0	0.0	0.0	0.0	0.0	0.0	0.0	0.0	0.0	0.0	0.0	0.0	0.0	0.0	0.0		100.0	99.8	100.0	100.0	100.0	100.0	100.0	100.0	100.0	100.0	100.0	99.8	100.0	100.0	100.0	100.0	100.0	99.8	100.0	18	UPT51768.1
19	0.0	0.0	0.0	0.0	0.0	0.0	0.0	0.0	0.0	0.0	0.0	0.0	0.0	0.0	0.0	0.0	0.0	0.0		99.8	100.0	100.0	100.0	100.0	100.0	100.0	100.0	100.0	100.0	99.8	100.0	100.0	100.0	100.0	100.0	99.8	100.0	19	QXP50053.1
20	0.2	0.2	0.2	0.2	0.2	0.2	0.2	0.2	0.2	0.2	0.2	0.2	0.2	0.2	0.2	0.2	0.2	0.2	0.2		99.8	99.8	99.8	99.8	99.8	99.8	99.8	99.8	99.8	100.0	99.8	99.8	99.8	99.8	99.8	100.0	99.8	20	UEN73261.1
21	0.0	0.0	0.0	0.0	0.0	0.0	0.0	0.0	0.0	0.0	0.0	0.0	0.0	0.0	0.0	0.0	0.0	0.0	0.0	0.2		100.0	100.0	100.0	100.0	100.0	100.0	100.0	100.0	99.8	100.0	100.0	100.0	100.0	100.0	99.8	100.0	21	QIA61508.1
22	0.0	0.0	0.0	0.0	0.0	0.0	0.0	0.0	0.0	0.0	0.0	0.0	0.0	0.0	0.0	0.0	0.0	0.0	0.0	0.2	0.0		100.0	100.0	100.0	100.0	100.0	100.0	100.0	99.8	100.0	100.0	100.0	100.0	100.0	99.8	100.0	22	AYW34065.1
23	0.0	0.0	0.0	0.0	0.0	0.0	0.0	0.0	0.0	0.0	0.0	0.0	0.0	0.0	0.0	0.0	0.0	0.0	0.0	0.2	0.0	0.0		100.0	100.0	100.0	100.0	100.0	100.0	99.8	100.0	100.0	100.0	100.0	100.0	99.8	100.0	23	QST88178.1
24	0.0	0.0	0.0	0.0	0.0	0.0	0.0	0.0	0.0	0.0	0.0	0.0	0.0	0.0	0.0	0.0	0.0	0.0	0.0	0.2	0.0	0.0	0.0		100.0	100.0	100.0	100.0	100.0	99.8	100.0	100.0	100.0	100.0	100.0	99.8	100.0	24	QBH90767.1
25	0.0	0.0	0.0	0.0	0.0	0.0	0.0	0.0	0.0	0.0	0.0	0.0	0.0	0.0	0.0	0.0	0.0	0.0	0.0	0.2	0.0	0.0	0.0	0.0		100.0	100.0	100.0	100.0	99.8	100.0	100.0	100.0	100.0	100.0	99.8	100.0	25	AXZ96151.1
26	0.0	0.0	0.0	0.0	0.0	0.0	0.0	0.0	0.0	0.0	0.0	0.0	0.0	0.0	0.0	0.0	0.0	0.0	0.0	0.2	0.0	0.0	0.0	0.0	0.0		100.0	100.0	100.0	99.8	100.0	100.0	100.0	100.0	100.0	99.8	100.0	26	QPB67620.1
27	0.0	0.0	0.0	0.0	0.0	0.0	0.0	0.0	0.0	0.0	0.0	0.0	0.0	0.0	0.0	0.0	0.0	0.0	0.0	0.2	0.0	0.0	0.0	0.0	0.0	0.0		100.0	100.0	99.8	100.0	100.0	100.0	100.0	100.0	99.8	100.0	27	QEY87900.1
28	0.0	0.0	0.0	0.0	0.0	0.0	0.0	0.0	0.0	0.0	0.0	0.0	0.0	0.0	0.0	0.0	0.0	0.0	0.0	0.2	0.0	0.0	0.0	0.0	0.0	0.0	0.0		100.0	99.8	100.0	100.0	100.0	100.0	100.0	99.8	100.0	28	UYW66597.1
29	0.0	0.0	0.0	0.0	0.0	0.0	0.0	0.0	0.0	0.0	0.0	0.0	0.0	0.0	0.0	0.0	0.0	0.0	0.0	0.2	0.0	0.0	0.0	0.0	0.0	0.0	0.0	0.0		99.8	100.0	100.0	100.0	100.0	100.0	99.8	100.0	29	WFV29811.1
30	0.2	0.2	0.2	0.2	0.2	0.2	0.2	0.2	0.2	0.2	0.2	0.2	0.2	0.2	0.2	0.2	0.2	0.2	0.2	0.0	0.2	0.2	0.2	0.2	0.2	0.2	0.2	0.2	0.2		99.8	99.8	99.8	99.8	99.8	100.0	99.8	30	YP_009703150.
31	0.0	0.0	0.0	0.0	0.0	0.0	0.0	0.0	0.0	0.0	0.0	0.0	0.0	0.0	0.0	0.0	0.0	0.0	0.0	0.2	0.0	0.0	0.0	0.0	0.0	0.0	0.0	0.0	0.0	0.2		100.0	100.0	100.0	100.0	99.8	100.0	31	WEH00179.1
32	0.0	0.0	0.0	0.0	0.0	0.0	0.0	0.0	0.0	0.0	0.0	0.0	0.0	0.0	0.0	0.0	0.0	0.0	0.0	0.2	0.0	0.0	0.0	0.0	0.0	0.0	0.0	0.0	0.0	0.2	0.0		100.0	100.0	100.0	99.8	100.0	32	WDZ17410.1
33	0.0	0.0	0.0	0.0	0.0	0.0	0.0	0.0	0.0	0.0	0.0	0.0	0.0	0.0	0.0	0.0	0.0	0.0	0.0	0.2	0.0	0.0	0.0	0.0	0.0	0.0	0.0	0.0	0.0	0.2	0.0	0.0		100.0	100.0	99.8	100.0	33	UUH61864.1
34	0.0	0.0	0.0	0.0	0.0	0.0	0.0	0.0	0.0	0.0	0.0	0.0	0.0	0.0	0.0	0.0	0.0	0.0	0.0	0.2	0.0	0.0	0.0	0.0	0.0	0.0	0.0	0.0	0.0	0.2	0.0	0.0	0.0		100.0	99.8	100.0	34	QOW03126.1
35	0.0	0.0	0.0	0.0	0.0	0.0	0.0	0.0	0.0	0.0	0.0	0.0	0.0	0.0	0.0	0.0	0.0	0.0	0.0	0.2	0.0	0.0	0.0	0.0	0.0	0.0	0.0	0.0	0.0	0.2	0.0	0.0	0.0	0.0		99.8	100.0	35	QTP96402.1
36	0.2	0.2	0.2	0.2	0.2	0.2	0.2	0.2	0.2	0.2	0.2	0.2	0.2	0.2	0.2	0.2	0.2	0.2	0.2	0.0	0.2	0.2	0.2	0.2	0.2	0.2	0.2	0.2	0.2	0.0	0.2	0.2	0.2	0.2	0.2		99.8	36	AJZ77059.1
37	0.0	0.0	0.0	0.0	0.0	0.0	0.0	0.0	0.0	0.0	0.0	0.0	0.0	0.0	0.0	0.0	0.0	0.0	0.0	0.2	0.0	0.0	0.0	0.0	0.0	0.0	0.0	0.0	0.0	0.2	0.0	0.0	0.0	0.0	0.0	0.2		37	QGV56826.1
	1	2	3	4	5	6	7	8	9	10	11	12	13	14	15	16	17	18	19	20	21	22	23	24	25	26	27	28	29	30	31	32	33	34	35	36	37		

Figure S2. Amino acid sequences analysis of p15 protein among different ASFV strains.
